# A comparison of RSV and influenza in vitro kinetic parameters reveals differences in infecting time

**DOI:** 10.1371/journal.pone.0192645

**Published:** 2018-02-08

**Authors:** Gilberto Gonzàlez-Parra, Filip De Ridder, Dymphy Huntjens, Dirk Roymans, Gabriela Ispas, Hana M. Dobrovolny

**Affiliations:** 1 Department of Physics and Astronomy, Texas Christian University, Fort Worth, TX, United States of America; 2 Department of Mathematics, New Mexico Tech, Socorro, NM, United States of America; 3 Janssen R&D Belgium, Beerse, Belgium; Mayo Clinic Minnesota, UNITED STATES

## Abstract

Influenza and respiratory syncytial virus (RSV) cause acute infections of the respiratory tract. Since the viruses both cause illnesses with similar symptoms, researchers often try to apply knowledge gleaned from study of one virus to the other virus. This can be an effective and efficient strategy for understanding viral dynamics or developing treatment strategies, but only if we have a full understanding of the similarities and differences between the two viruses. This study used mathematical modeling to quantitatively compare the viral kinetics of in vitro RSV and influenza virus infections. Specifically, we determined the viral kinetics parameters for RSV A2 and three strains of influenza virus, A/WSN/33 (H1N1), A/Puerto Rico/8/1934 (H1N1), and pandemic H1N1 influenza virus. We found that RSV viral titer increases at a slower rate and reaches its peak value later than influenza virus. Our analysis indicated that the slower increase of RSV viral titer is caused by slower spreading of the virus from one cell to another. These results provide estimates of dynamical differences between influenza virus and RSV and help provide insight into the virus-host interactions that cause observed differences in the time courses of the two illnesses in patients.

## Introduction

Acute respiratory tract infections with respiratory syncytial virus (RSV) and influenza are leading causes of respiratory illness [1]. Both infections produce similar symptoms and lead to serious illness primarily in the young and the elderly [2, 3]. Given these similarities, it can be useful to compare the viral dynamics of the two viruses in cells because this may help to understand the different viral dynamics in patients, and consequently to translate the knowledge of treating one illness to help treating the other.

Comparison of the two illnesses began shortly after the discovery of RSV [4]. Until recently, comparative studies focused on the mortality of the two diseases [5], to understand disease burden, and on differentiating the symptoms of the two diseases [4, 6–8], in order to assist with diagnosis. More recent comparative studies have turned to investigations of differences in immune response [8–11] in an effort to more fundamentally understand dynamical differences between the two diseases. Of particular interest is a recent study by Bagga *et al*. [12] in which healthy volunteers were inoculated with either influenza virus or RSV and daily viral loads were measured. A comparison of the viral titer curves noted that RSV showed a longer incubation period than influenza virus. Influenza virus also appeared to propagate very quickly, rising from first detection to peak viral titer within 24 h, while RSV grew more slowly, taking between 24–48 h to reach the peak viral titer. Both of these features lead to a later time of peak viral titer for RSV than for influenza virus. Since this was a clinical study, with no observation of the infection at the cellular level, the underlying mechanism for slower RSV growth was not determined. The authors themselves suggested that a better understanding of the cellular-level mechanisms causing differences between RSV and influenza virus could help in the development of antivirals for RSV.

There currently are antivirals for treatment of influenza virus and promising new antivirals in the pipeline [13, 14]. However, there has historically been less success in development of antivirals for RSV [15, 16]. A few possible RSV antivirals are being investigated [17–19], but a better understanding of the similarities and differences between RSV and influenza virus might allow development of more effective antivirals against both infections.

Mathematical models of viral infections can help us develop a better understanding of differences in viral propagation dynamics through a quantitative comparative analysis of viral kinetics parameters. This type of analysis has previously been done to compare different strains of influenza virus [20–22]. Influenza viral kinetics models have also helped further our understanding of the causes of disease severity [23], the emergence of drug resistance [24, 25], and intracellular viral replication [26]. While more complex, some attempts have been made to extend these models to reflect in vivo infections through the inclusion of an immune response [27–29] or consideration of associated symptoms [25, 30]. In addition, mathematical models can be used to describe the pharmacodynamic effects of new compounds in a strict quantitative manner [31, 32]. Thus, the combination of viral kinetics and pharmacodynamic models can help us assess the influence of different mechanisms of actions [26, 33], or the effect of combination therapies [34].

In this paper, we describe a quantitative comparative analysis of RSV and influenza viral kinetics. We use data collected from literature of in vitro RSV and influenza virus infections to extract viral kinetics parameters through fitting with both an empirical and a viral kinetics model. We then use statistical analysis to determine whether there are significant differences in viral kinetics between RSV and influenza virus. Our results support the findings of Bagga et. al. [12] since we find that RSV has a slower growth rate and later time of viral peak than influenza virus in vitro. Our analysis, however, also suggests a possible mechanism for these observations since we find that the key difference in the dynamics of RSV and influenza virus is that RSV takes longer to transmit virus from infectious cells to healthy cells.

## Materials and methods

### Models

We use two models to characterize both RSV and influenza virus in vitro infections. The first model is an empirical description of the viral time course, first presented by Holder and Beauchemin in 2011 [35]. While this model does not give insight into the underlying dynamics of the infection, it allows for a quantitative comparison of viral titer characteristics between influenza virus and RSV. The model is given by the equation

$$\begin{array}{c} V\left(t\right)=\frac{2V_{p}}{\exp\left[-\lambda_{g}\left(t-t_{p}\right)\right]+\exp\left[\lambda_{d}\left(t-t_{p}\right)\right]}, \end{array}$$ (1)

where λ_*g*_ and λ_*d*_ are the exponential growth and decay rates, respectively, *V*_*p*_ is the peak viral titer, and *t*_*p*_ is the time of viral titer peak. Note that the growth and decay rates here refer to growth and decay in the number of viral particles. This simple functional form has only four independent parameters, making parameter identifiability simpler than for a kinetic model of viral infection. For this model, four data points are required, with at least two data points required during the growth phase and at least two required during the decay phase to identify the parameters.

The second model we will use is a viral kinetics model. The model is an extension of the basic viral infection model for influenza virus described in [36],

$$\begin{array}{ccc} \frac{dT}{dt} & = & -\frac{\beta }{N}TV\\ \frac{dE_{1}}{dt} & = & \frac{\beta }{N}TV-\frac{n_{E}}{\tau_{E}}E_{1}\\ \frac{dE_{j}}{dt} & = & \frac{n_{E}}{\tau_{E}}E_{j-1}-\frac{n_{E}}{\tau_{E}}E_{j}\;\;\;\text{for}\;j=\left(2,\ldots ,n_{E}\right)\\ \frac{dI_{1}}{dt} & = & \frac{n_{E}}{\tau_{E}}E_{n_{E}}-\frac{n_{I}}{\tau_{I}}I_{1}\\ \frac{dI_{j}}{dt} & = & \frac{n_{I}}{\tau_{I}}I_{j-1}-\frac{n_{I}}{\tau_{I}}I_{j}\;\;\;\text{for}\;j=\left(2,\ldots ,n_{I}\right)\\ \frac{dV}{dt} & = & p\sum_{j=1}^{n_{I}}I_{j}-cV\;. \end{array}$$ (2)

**Fig 1:**
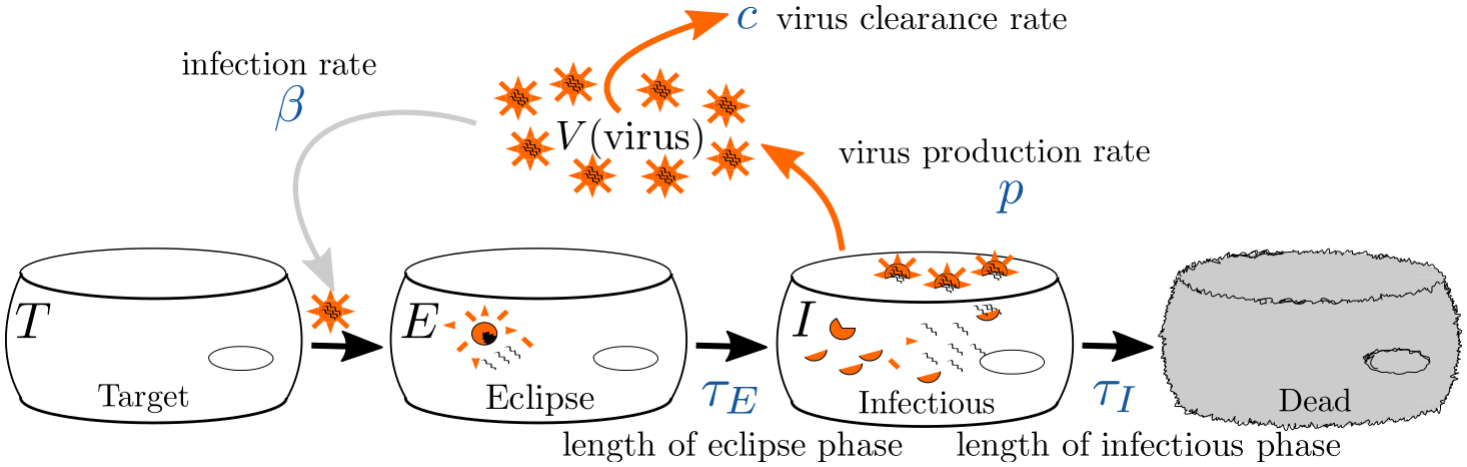
Viral kinetics model. The virus, *V*, attacks target cells, *T*, at rate *β*. Once infected, target cells enter the eclipse phase, *E*. The eclipse phase lasts an average time of *τ*_*E*_, after which the cells become infectious cells, *I*. The infectious cells produce new virions at rate *p*, and the virus decays at rate *c*. The cells remain infectious for an average time of *τ*_*I*_, after which they die.

In the model, pictured in Fig 1, target cells, *T*, become infected at rate *β* when they encounter virus, *V*. Upon infection, the cells enter an eclipse state, *E*, where they are infected, but not yet producing virus. After an average time *τ*_*E*_, the cells transition to a productively infectious state, *I*, where they are producing virus at rate *p*. After an average time *τ*_*I*_, the infected cells die. Virus loses infectivity at a rate *c*. Since our model does not explicitly include an adaptive immune response, the value of *c* will also reflect the contribution of the in vitro innate response in clearing the virus. Studies indicate, however, that in vitro loss of viral infectivity for influenza virus is primarily due to degradation of the virus rather than the effect of an immune response [20, 37]. This model assumes a gamma distribution, represented by the multiple compartments for *E* and *I*, for the transition times between the eclipse state and the infectious state, as well as for the transition times between the infectious and dead cells. The number of compartments in the eclipse state is given by *n*_*E*_ while the number of compartments in the infectious state is given by *n*_*I*_. Gamma-distributed models of viral kinetics have previously been used to study influenza virus infections [20, 21], as well as SHIV infections [38]. This model has more parameters than the empirical model, some of which cannot be identified with viral titer data alone [20, 39].

### Data selection criteria

The literature was searched for data of in vitro RSV and influenza virus infections. Only data from studies with a reported multiplicity of infection (MOI) of less than one, were used. We further required that the studies measure released virus or virus in the supernatent rather than cell-associated virus. A third requirement was that the experimental data contained at least two data points during the growth phase of the infection and two data points during the decay phase of the infection, so that parameters from both phases of the infection could be estimated. For RSV data, all data sets use the A2 strain of RSV, but since there is wide variation in the cell lines used for infections, only studies that used cells of human origin were included. For influenza virus, data was limited to the following common experimental strains: A/WSN/33 (H1N1), which was denoted WSN33; the H1N1 pandemic strain, which was denoted pH1N1; and A/Puerto Rico/8/1934 (H1N1), which was denoted PR8. For influenza virus, experiments performed in Madin-Darby canine kidney (MDCK) cells were selected, since this is the most common experimental substrate for influenza virus experiments. In addition, a number of in vitro experiments of WSN33 infections in Madin-Darby bovine kidney (MDBK) cells were included in our study, which were used to examine the effect of cell type on viral kinetics parameters.

### Fitting algorithms

The model was fitted to in vitro viral RSV and influenza virus experimental data in order to obtain the parameter value estimates for each virus. We determined the best fit by minimizing the sum of squared residuals (SSR),

$$\begin{array}{c} \text{SSR}=\sum_{i=1}^{n}{\left(y_{i}-f\left(t_{i}\right)\right)}^{2}, \end{array}$$ (3)

where *n* is the number of experimental data points, *y*_*i*_ are the values of the experimental data points, and *f*(*t*_*i*_) are the model predictions at the times when experimental data were measured. A small SSR indicates a tight fit of the model to the experimental data. In order to better compare fits of the models between different data sets, we also calculated the root mean squared error,

$$\begin{array}{c} \sqrt{\text{MSE}}=\sqrt{\frac{\text{SSR}}{n-N}}, \end{array}$$ (4)

where *n* is again the number of data points and *N* is the number of free parameters in the model. $\sqrt{\text{MSE}}$ corrects for differences in number of data points between different data sets, providing a more standardized measure of goodness of fit. Note that $\sqrt{\text{MSE}}$ does not exist if the number of data points is less than the number of parameters *n* < *N*.

We used the fmincon algorithm in Matlab to find the minimum SSR for both the empirical and viral kinetics models. For the empirical model, no constraints were used in the fitting process. For the viral kinetics model, we fixed the initial number of target cells to *T*_0_ = 1 and assumed that the infection was started with an unknown (to be fitted) initial viral inoculum, assuming that there are initially no cells in any of the eclipse or infectious compartments. We know that some parameters are not identifiable for this model [20, 39], so we fixed the number of compartments in both the eclipse and infectious phases, *n*_*I*_ = *n*_*E*_ = 60, and set bounds on the searched parameter space as follows: 10–10^10^ /h for *p*; 10^−10^–10 /h for *β*; 10^−4^–10 /h for *c*; 2–72 h for *τ*_*I*_; 1–48 h for *τ*_*E*_; 10^−7^–10^16^ for *V*_0_. Note that the bounds are quite large and are meant to eliminate the possibility of finding biologically unrealistic parameter values.

### Viral kinetics parameters

It is difficult to compare parameter estimates from different experiments since the units of viral titer depend on the assay used to determine the viral titer, as well as viral extraction and amplification methods [21]. There is no universal standard viral titer unit, making comparison of parameters such as *p* and *β* irrelevant. Therefore, we focused on parameters which have a universal standardized unit. In addition to the mean duration of the eclipse phase *τ*_*E*_ and the mean duration of the infectious phase *τ*_*I*_, the infecting time, $t_{\text{inf}}=\sqrt{2/p\beta }$, [35], which is the average time between release of a virion from an infectious cell and infection of another cell, was calculated. The infecting time can be derived directly from the viral kinetics model equations as follows. We assume that we have a single infected cell and that viral degradation is negligible during this short time frame, so the viral equation becomes

$$\begin{array}{c} \frac{dV}{dt}=p. \end{array}$$

This is easily integrated to give

$$\begin{array}{c} V(t)=pt. \end{array}$$

Since we are interested in counting cells entering the eclipse phase, and not those leaving, the equation for eclipse cells becomes

$$\begin{array}{c} \frac{dE}{dt}=\frac{\beta }{N}VT. \end{array}$$

Substituting our result for *V* into this equation and assuming that *T* ≈ *N*, the differential equation for *E* becomes

$$\begin{array}{c} \frac{dE}{dt}=\beta pt. \end{array}$$

The infecting time is the time at which a single new cell is infected, so we integrate *E* from 0 to 1 and *t* from 0 to *t*_inf_,

$$\begin{array}{c} 1=\beta p\frac{t_{\text{inf}}^{2}}{2}, \end{array}$$

or solving for infecting time,

$$\begin{array}{c} t_{\text{inf}}=\sqrt{\frac{2}{p\beta }}. \end{array}$$

### Statistical analysis

In order to identify statistically significant differences in parameters between relevant groups, we performed a Mann-Whitney (Wilcoxon rank-sum) test. We use the Mann-Whitney test since we cannot assume normal distributions for the parameters as is required for other statistical tests. When distributions are continuous, as they are in our case, the Mann-Whitney test can be interpreted as determining whether there is a significant difference in the medians of the two distributions. In our analysis, the Mann-Whitney test was used to determine whether the median of a given parameter obtained by fitting one set of data (RSV, for example) was equal to that obtained by fitting another set of data (one of the flu data sets).

## Results

### Comparison of RSV and influenza virus

**Fig 2:**
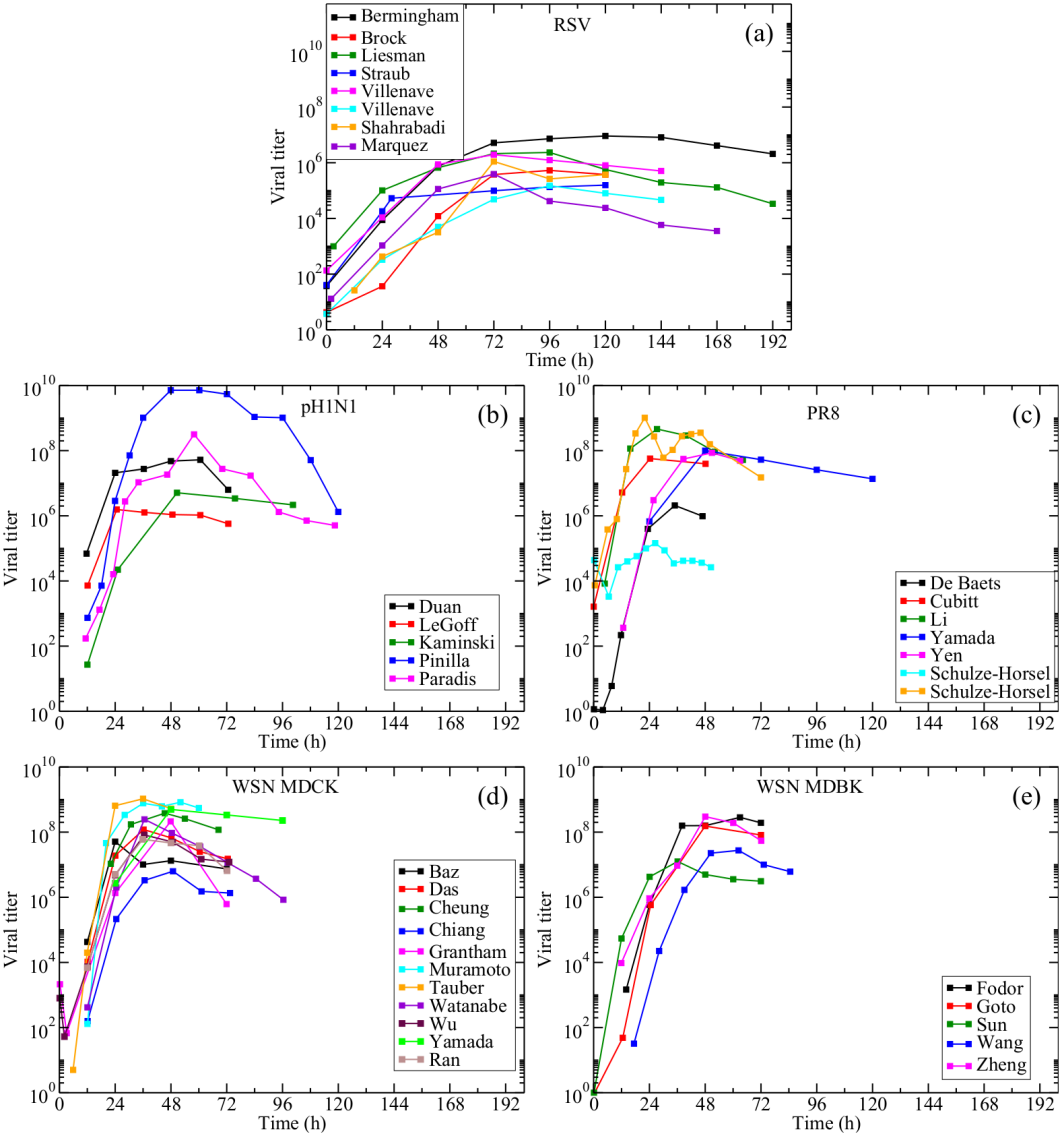
Study data. In vitro RSV and influenza virus infection data used in this study.

We searched the literature and collected 36 data sets of experimental infections that satisfied the requirements outlined in the Methods section. A summary of the data sets used in this study is given in Table 1. The data sets are shown, in groups, in Fig 2. The RSV data sets were fairly consistent, showing similar growth rates and time of peak viral titer (Fig 2a). The pH1N1 data sets also showed similar growth rates, but seemed to vary in their time and extent of peak viral titer (Fig 2b). Moreover, most data sets showed a plateau of the viral titer probably because all cells in the cultures were infected at the moment peak viral titer was reached. The PR8 data sets seem to have the fastest growth, with the exception of one of the Schulze-Horsel data sets (Fig 2c). The WSN in MDCK data sets also show similar growth rates, although the peak viral titer varies. (Fig 2d). The variation in peak viral titer is somewhat less when WSN infects MDBK cells, although there seems to be more variation in the growth rate of WSN in MDBK cells (Fig 2e).

**Table 1:** Data sets used in this study.

Paper	Figure*	Cell type	Number of data points	MOI
RSV				
Bermingham (1999) [40]	4B	HEp-2	9	0.01
Brock (2003) [41]	1B	HEp-2	6	0.25
Liesman (2014) [42]	1C	HAE	9	1
Marquez (1967) [43]	2	HEp-2	8	0.01
Shahrabadi (1988) [44]	2A	HEp-2	6	0.1
Straub (2011) [45]	2A	A549	6	0.1
Villenave (2011) [46]	4A (A2 strain)	PBEC	5	0.1
Villenave (2012) [47]	1A (A2 strain)	WD-PBEC	7	0.1
pH1N1				
Duan (2010) [48]	1C	MDCK	6	0.001
Kaminski (2013) [49]	2A	MDCK	5	0.001
Le Goff (2012) [50]	4	MDCK	6	0.001
Pinilla (2012) [20]	2A	MDCK	12	5 × 10^−5^
Paradis (2015) [21]	1A	MDCK	12	5 × 10^−5^
PR8				
Cubitt (1997) [51]	3B	MDCK	4	0.5
De Baets (2013) [52]	1E	MDCK	7	0.001
Li (2010) [53]	2B	MDCK	6	0.001
Schulze-Horsel (2009) [54]	3A	MDCK	13	0.025
Schulze-Horsel (2009) [54]	3B	MDCK	14	0.025
Yamada (2012) [55]	2	MDCK	5	0.001
Yen (2007) [56]	1D	MDCK	5	1 × 10^−4^
WSN33 MDCK				
Baz (2010) [57]	1B	MDCK	5	0.001
Cheung (2005) [58]	3B	MDCK	5	0.01
Chiang (2008) [59]	7A	MDCK	6	0.001
Das (2012) [60]	1D	MDCK	6	0.001
Grantham (2009) [61]	1B	MDCK	5	0.001
Muramoto (2013) [62]	3	MDCK	7	5 × 10^−4^
Ran (2013) [63]	2	MDCK	6	0.001
Tauber (2012) [64]	3	MDCK	5	0.001
Watanabe (2003) [65]	6	MDCK	8	0.001
Wu (2008) [66]	1B	MDCK	8	0.001
Yamada (2012) [55]	2	MDCK	4	0.001
WSN33 MDBK				
Fodor (1998) [67]	2	MDBK	6	0.01
Goto (2001) [68]	1	MDBK	5	1 × 10^−4^
Sun (2010) [69]	2B	MDBK	7	0.01
Wang (2002) [70]	1B	MDBK	7	0.001
Zheng (1996) [71]	6	MDBK	6	0.001

* Refers to the figure numbers in the original paper.

**Fig 3:**
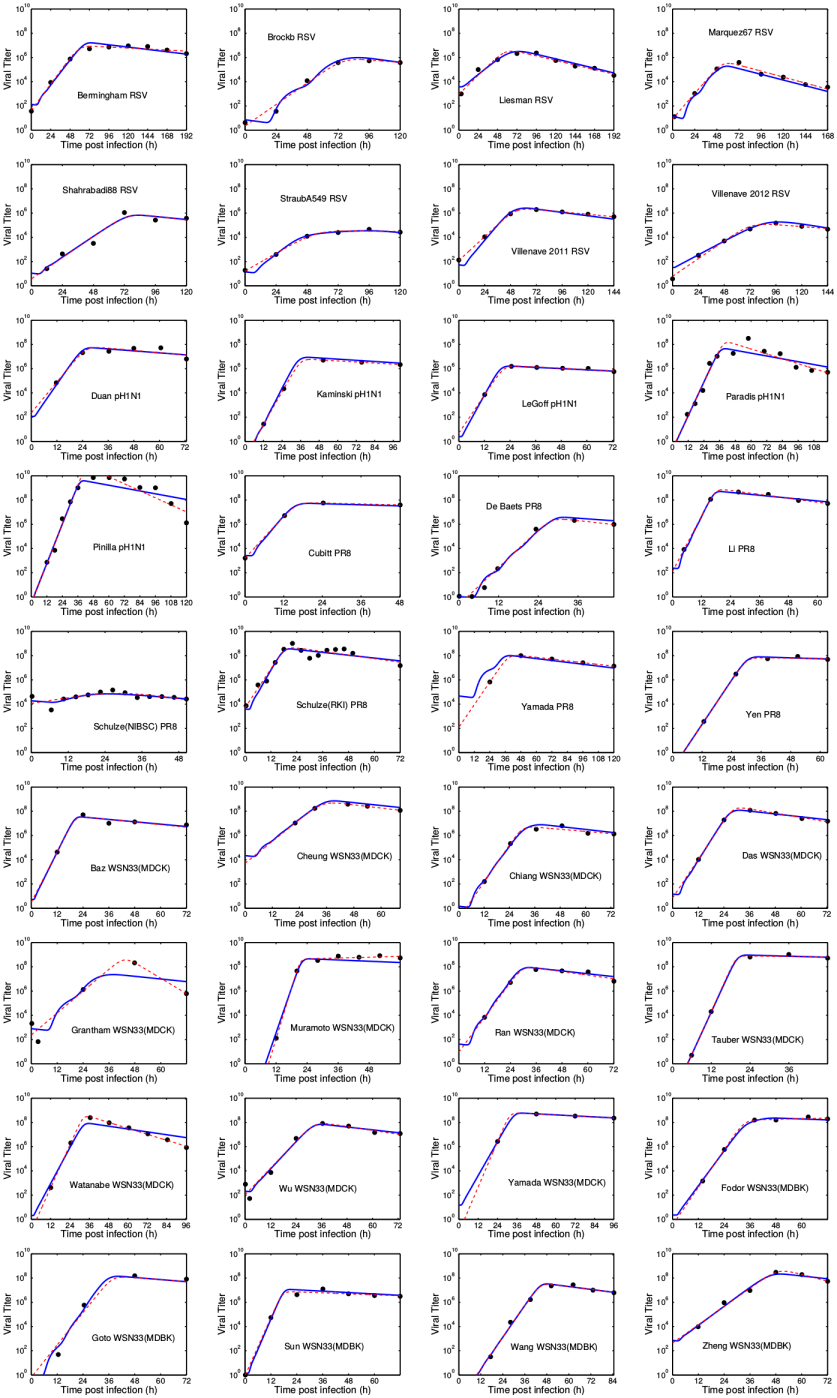
Empirical and viral kinetics model fits. We fit both the empirical model (Eq (1)) and the viral kinetics model (Eq (2)) to each of the RSV and influenza virus viral time courses. The best fit curves are shown in Fig 3 where the red dashed line represents the best fit empirical model and the blue solid line represents the best fit viral kinetics model. The extracted parameter values are presented in Table 2 for the empirical model and in Table 3 for the viral kinetics model.

**Fig 4:**
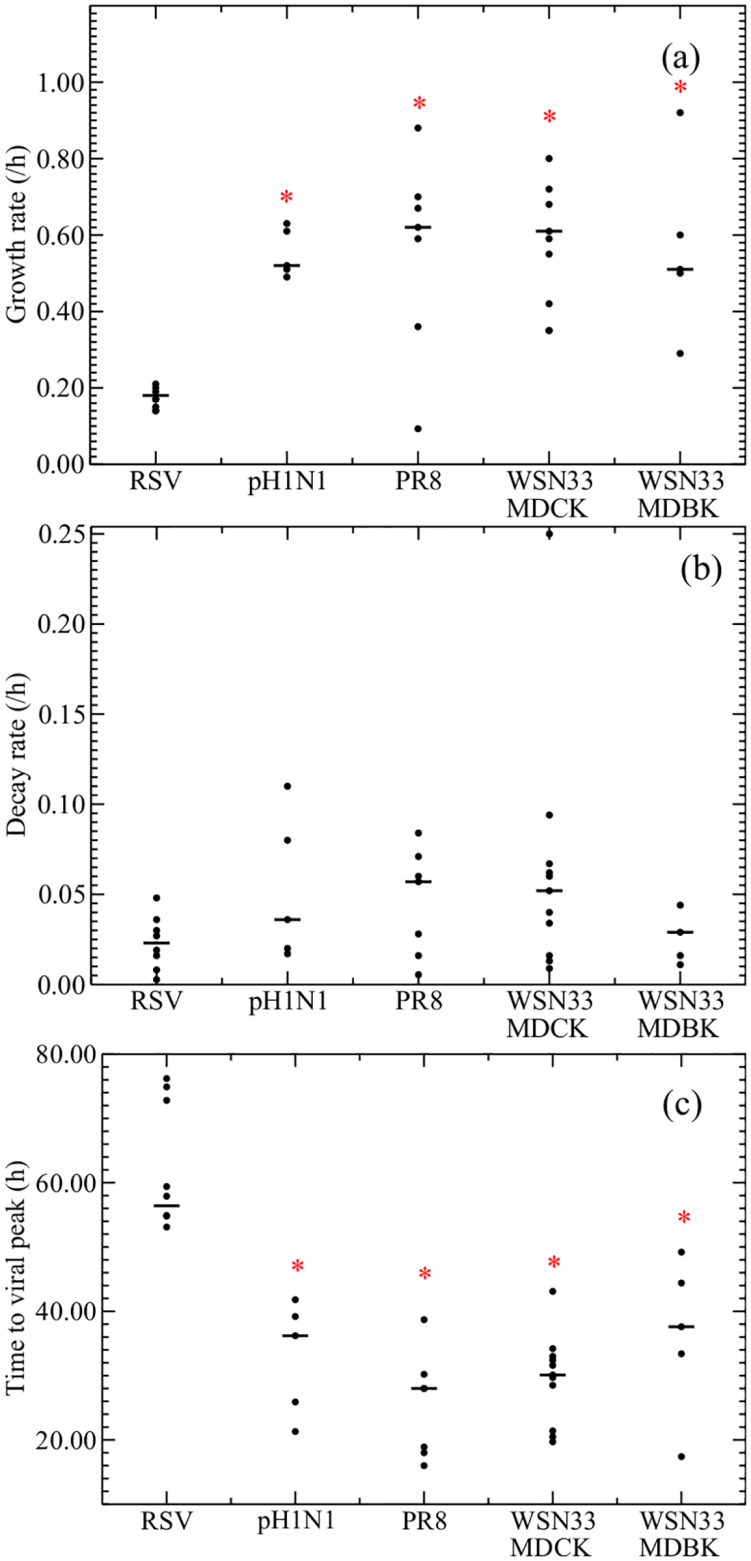
Comparison of empirical model parameters. Graphs show the distributions of (a) growth rate, (b) decay rate, (c) time to peak viral titer estimated from fits of the empirical model for RSV and the different strains of influenza virus. Statistically significant differences (*p* < 0.05) between RSV and a particular influenza virus strain are indicated with an asterisk. The *p*-values for the Mann-Whitney test are given in Table 4. Median values are indicated with a solid black line.

Since the empirical model helps to quantitatively describe the temporal course of viral titer, we first fitted all the selected RSV and influenza virus data sets to this model (Fig 3). The analysis demonstrated that the median growth rate for RSV is 0.18 IU/h, while for different influenza virus strains this is significantly faster, ranging from 0.51 to 0.62 IU/h (Table 2, Fig 4), meaning that influenza viral titer grows about three times more rapidly than RSV viral titer. The median decay rate for RSV was calculated to be 0.023 IU/h and was found to be similar for the different influenza virus strains (0.029 IU/h − 0.057 IU/h) (Table 2, Fig 4). Our data analysis demonstrates that infections with different influenza virus strains reach time of peak viral titer about one day earlier than RSV.

**Table 2:** Estimated parameter values for the empirical model (Eq (1)).

Data	*V*_*p*_	λ_*g*_ (IU/h)	λ_*d*_ (IU/h)	*t*_*p*_ (h)	SSR	$\sqrt{\text{MSE}}$
Bermingham	4.91 × 10^6^	0.21	0.0081	59.4	0.194	0.20
Brock	4.71 × 10^5^	0.17	0.027	76.2	0.433	0.47
Liesman	2.72 × 10^6^	0.15	0.036	57.9	0.398	0.28
Marquez	2.67 × 10^5^	0.20	0.048	54.8	0.156	0.20
Shahrabadi	5.19 × 10^5^	0.17	0.030	74.9	0.716	0.60
Straub	1.84 × 10^4^	0.14	0.0027	54.9	0.057	0.17
Villenave 2011	1.45 × 10^6^	0.19	0.019	53.1	0.003	0.055
Villenave 2012	8.15 × 10^4^	0.14	0.016	72.8	0.182	0.25
RSV median	3.93 × 10^5^	0.18	0.023	56.4	0.169	0.23
Duan	3.59 × 10^7^	0.49	0.036	25.9	0.36	0.42
Kaminski	3.30 × 10^6^	0.51	0.017	36.2	1.05 × 10^−04^	0.010
LeGoff	9.38 × 10^5^	0.61	0.020	21.3	0.015	0.087
Paradis	1.06 × 10^8^	0.52	0.08	39.2	2.34	0.54
Pinilla	3.19 × 10^10^	0.63	0.11	41.8	2.79	0.59
pH1N1 median	3.59 × 10^7^	0.52	0.036	36.2	0.36	0.42
Cubitt*	3.26 × 10^7^	0.67	0.016	16.0	1.04 × 10^−12^	–
de Baets	1.80 × 10^6^	0.59	0.071	28.0	0.97	0.57
Li	4.53 × 10^8^	0.88	0.06	18.0	0.015	0.087
Schulze 3a	7.68 × 10^4^	0.093	0.084	28.0	1.18	0.36
Schulze 3b	2.91 × 10^8^	0.62	0.057	18.9	1.58	0.40
Yamada	6.69 × 10^7^	0.36	0.028	38.7	2.1 × 10^−4^	0.014
Yen	3.44 × 10^7^	0.70	0.0054	30.2	0.031	0.18
PR8 median	3.44 × 10^7^	0.62	0.057	28.0	0.031	0.27
Baz	1.98 × 10^7^	0.80	0.04	20.5	0.15	0.39
Cheung	3.50 × 10^8^	0.35	0.052	34.2	2.7 × 10^−4^	0.016
Chiang	3.02 × 10^6^	0.59	0.034	30.1	0.13	0.25
Das	1.28 × 10^8^	0.61	0.067	28.5	0.092	0.21
Grantham	3.66 × 10^8^	0.35	0.25	43.1	1.9	1.4
Muramoto	2.19 × 10^8^	1.61	0.013	21.4	0.074	0.16
Ran	5.87 × 10^7^	0.55	0.06	29.7	0.13	0.25
Tauber	4.11 × 10^8^	1.37	8.9 × 10^−3^	19.7	0.045	0.21
Watanabe	2.20 × 10^8^	0.68	0.094	32.4	0.026	0.080
Wu	5.85 × 10^7^	0.42	0.062	33.0	1.5	0.62
Yamada*	3.24 × 10^8^	0.72	0.016	31.6	1.0 × 10^−12^	–
WSN33 MDCK median	2.19 × 10^8^	0.61	0.052	30.1	0.092	0.23
Fodor	7.86 × 10^7^	0.60	-0.01	33.4	0.026	0.11
Goto	7.14 × 10^7^	0.51	0.029	37.6	0.11	0.33
Sun	4.00 × 10^6^	0.92	0.016	17.4	0.16	0.23
Wang	1.94 × 10^7^	0.50	0.044	44.4	0.20	0.62
Zheng	3.28 × 10^8^	0.29	0.011	49.2	0.16	0.28
WSN33 MDBK median	7.14 × 10^7^	0.51	0.029	37.6	0.16	0.28

* indicates data sets with only 4 data points where $\sqrt{\text{MSE}}$ is undefined.

**Fig 5:**
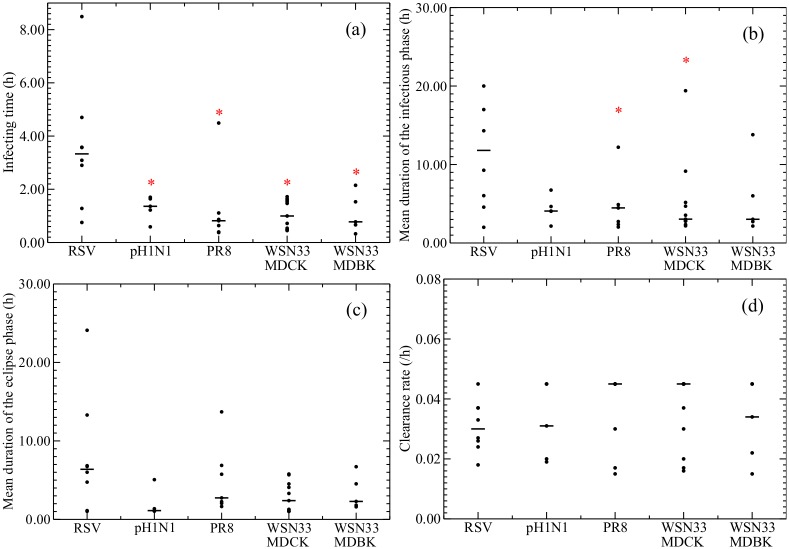
Comparison of viral kinetics parameters. Graphs show the distributions of (a) infecting time (*t*_inf_), (b) duration of the infectious phase (*τ*_*I*_), (c) duration of the eclipse phase (*τ*_*E*_), and (d) degradation rate (*c*) estimated from fits of the gamma model for RSV and the different strains of influenza virus. Statistically significant differences (*p* < 0.05) between RSV and a particular influenza virus strain are indicated with an asterisk. The *p*-values for the Mann-Whitney test are given in Table 4. Median values are indicated with a solid black line.

**Fig 6:**
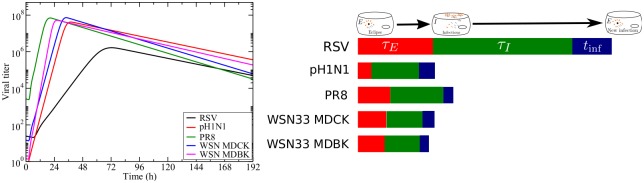
Differences in infection time course. (a) Predicted time courses of RSV, pH1N1, PR8, WSN33 MDCK, and WSN33 MDBK using the median values (Table 3) for each of the parameters in the viral kinetics model (Eq (2)). (b) A schematic diagram of the duration of different phases (using median values) of the viral replication cycle for RSV, pH1N1, PR8, WSN33 MDCK, and WSN33 MDBK.

Next, data from the same set of studies were analysed by the viral kinetics model to obtain some insight into the processes governing the viral life cycle (Table 3 & Fig 3). With this analysis, we estimated *t*_inf_, the time between release of a virus from one cell and infection of the next cell; *c*, the degradation rate; *τ*_*I*_, the duration of the infectious phase; and *τ*_*E*_, the duration of the eclipse phase. We determined that the median *t*_inf_ for RSV of 3.00 h is considerably longer than for influenza virus (Tables 3 & 4, Fig 5). Degradation rates appear to be similar for RSV and different influenza virus strains with median values ranging from 0.030 IU/h to 0.045 IU/h. These values are similar to the values found for the decay rate using the empirical model. The median duration of the infectious phase for RSV is 11.8 h, which is longer than the 3 to 4 h median values found for the influenza virus data sets. Finally, the median duration of the eclipse phase for RSV (6.38 h) is also longer than the median duration found for the influenza virus data sets (between 1.1 and 2.7 h). Combined, these data can be used to model the time course of RSV and different influenza virus strains (Fig 6a), and they explain why RSV reaches peak viral titer later than influenza virus (Fig 6b).

**Table 3:** Estimated parameter values for the viral kinetics model (Eq (2)).

Data	*t*_inf_ (h)	*c* (IU/h)	*τ*_*I*_ (h)	*τ*_*E*_ (h)	SSR	$\sqrt{\text{MSE}}$
Bermingham	2.90	0.018	6.03	6.84	0.734	0.49
Brock*	0.753	0.033	20.0	24.1	0.268	–
Liesman	8.49	0.037	14.3	1.12	0.645	0.47
Marquez	1.28	0.045	9.27	13.3	0.508	0.50
Shahrabadi*	3.57	0.024	4.57	6.01	0.710	–
Straub*	3.58	0.026	66.8	6.74	0.037	–
Villenave 2011*	3.09	0.027	17.0	4.75	0.310	–
Villenave 2012	4.70	0.037	2.00	1.03	0.984	0.99
RSV median	3.33	0.030	11.8	6.38	0.576	0.50
Duan*	1.64	0.031	4.04	1.07	0.437	–
Kaminski*	0.589	0.019	6.74	5.06	0.164	–
LeGoff*	1.22	0.020	4.07	1.12	0.018	–
Paradis	1.70	0.045	4.65	1.36	3.14	0.72
Pinilla	1.36	0.045	2.15	1.05	6.07	1.0
pH1N1 median	1.36	0.031	4.07	1.12	0.437	0.86
Cubitt*	0.818	0.017	4.47	2.26	0.057	–
de Baets	0.374	0.045	2.03	5.75	0.760	0.87
Li*	0.397	0.045	2.36	2.74	0.074	–
Schulze 3a	4.49	0.045	12.2	6.88	0.740	0.33
Schulze 3b	0.867	0.045	2.73	2.04	1.84	0.48
Yamada*	0.636	0.030	4.89	13.7	1.06	–
Yen*	1.11	0.015	4.48	1.64	0.038	–
PR8 median	0.818	0.045	4.47	2.74	0.740	0.48
Baz*	0.996	0.037	2.18	1.04	0.154	–
Cheung*	1.47	0.045	2.88	4.52	0.125	–
Chiang*	0.538	0.045	9.15	5.78	0.245	–
Das*	0.718	0.045	5.17	3.31	0.040	–
Grantham*	1.64	0.030	19.4	4.08	3.78	–
Muramoto	0.482	0.020	2.78	1.01	0.854	0.92
Ran*	0.474	0.045	4.69	5.68	0.204	–
Tauber*	0.449	0.017	2.79	1.28	0.054	–
Watanabe	1.53	0.045	2.35	1.04	1.38	0.83
Wu	1.59	0.045	3.04	2.39	0.885	0.66
Yamada*	1.72	0.016	3.54	1.22	0.001	–
WSN33 MDCK median	0.996	0.045	3.04	2.39	0.204	0.83
Fodor*	1.53	0.015	13.8	1.81	0.088	–
Goto*	0.326	0.034	6.01	6.71	0.603	–
Sun	0.663	0.022	2.72	1.60	0.272	0.52
Wang	0.777	0.045	3.02	4.53	0.199	0.47
Zheng*	2.15	0.045	2.16	2.29	0.246	–
WSN33 MDBK median	0.777	0.034	3.02	2.29	0.246	0.50

* indicates data sets with 6 or less data points where $\sqrt{\text{MSE}}$ is undefined.

**Table 4:** Results of the Mann-Whitney tests (p-values).

	λ_*g*_	λ_*d*_	*t*_*p*_	*t*_inf_	*τ*_*I*_	*τ*_*E*_	*c*
Comparing RSV and influenza virus strains							
pH1N1	**0.0034**	0.19	**0.013**	**0.047**	0.079	0.079	0.83
PR8	**0.021**	0.16	**0.0018**	**0.028**	**0.049**	0.64	0.52
WSN33 MDCK	**2.8 × 10^−4^**	0.058	**3.8 × 10^−4^**	**0.0064**	**0.048**	0.058	0.32
WSN33 MDBK	**0.0034**	0.56	**0.013**	**0.0228**	0.11	0.24	0.94
Comparing different influenza virus strains							
pH1N1/ PR8	0.46	0.68	0.17	0.17	0.94	**0.028**	0.87
pH1N1/ WSN33	0.40	0.87	0.40	0.40	0.77	0.61	0.69
PR8 / WSN33	0.75	0.89	0.13	0.50	0.82	0.19	0.89
Comparing the effect of cell type							
MDCK / MDBK	0.40	0.46	0.13	0.95	0.87	0.40	0.46

### Comparison of different influenza virus strains

Since we determined viral kinetics parameters for several different strains of influenza virus, influenza virus strains can be compared. Our analysis demonstrates that the estimated parameters for different strains of influenza virus are quite consistent. We found no statistically significant differences between the parameters of the different influenza virus strains except that the median duration of the pH1N1 eclipse phase is lower than the median eclipse phase of PR8 (Fig 5). Given the recent finding that viral kinetics parameter estimates can vary substantially between experiments using the same strain in the same cell line [21], we see a remarkably good consistency in parameter estimates from different strains of influenza virus extracted from different experiments.

### The effect of cell type

Because it is well-known that the host cell line may have a major influence on the propagation of a virus infection [72–74], we investigated this effect by analysing the data of influenza virus studies in MDCK and MDBK cells. Our analysis demonstrated that the parameter values for WSN33 in either cell type are likely from the same distributions, suggesting that there is little difference between influenza virus infections in either MDCK or MDBK cells.

## Discussion

### Causes of the slower growth of RSV

This paper quantitatively examines the differences between in vitro RSV and influenza virus infection experiments. We found that RSV has a statistically significantly slower growth rate and later time to reach viral titer peak than influenza virus. While this was previously observed clinically [12], our study indicates that there are similar differences in vitro. Our use of a viral kinetics model also allowed examination of possible mechanisms for this observed difference. This analysis determined that RSV has a significantly longer infecting time (*t*_inf_) than influenza virus, providing a possible dynamical explanation for the observed slower growth rate. Since RSV takes longer to spread from an infectious cell to a new target cell, this will lead to an overall slower growth rate. While not statistically significant, the duration of the eclipse phase was also longer in RSV than in flu, which could also contribute to the slower growth of RSV. Finally, we found that RSV has a longer infectious lifespan than PR8 or WSN33 in MDCK cells, which could contribute to the longer propagation of RSV infection.

Our findings help explain some observations made during the in vivo comparison of influenza virus and RSV infections [12]. The slower growth rate and delayed time of peak titer in vivo are mirrored by a slower growth rate and delayed time to reach peak viral titer in vitro, suggesting that these features are not due to adaptive immune interactions of either virus, but are most likely a consequence of virus-cell interactions. Note that time of peak is known to depend on the initial viral inoculum [75], with lower MOI leading to a later time of peak. The influenza virus data used in this study uses a lower MOI than RSV, suggesting that the difference in time of peak viral titer would be greater if both infections were initiated with the same inoculum.

There are two fundamental processes included in the infecting time, movement of the virus from its originating cell to a new target cell and entry of the virus into the new target cell. While new imaging modalities allow tracking of virus particles during an infection [76–78], they have largely been used to track virions after they enter a target cell. One study, however, examined motion of swine influenza virus in porcine respiratory mucus finding that most of the virions (∼70%) were immobilized in mucus, but the remaining 30% that traveled, moved through diffusion [79]. Thus comparison of diffusion coefficients might give an estimate of which virus is likely to travel between cells faster. RSV is twice as big as influenza virus (120–220 nm [80] versus 80–120 nm [81]), so we expect influenza virus to have a larger diffusion coefficient simply due to its smaller size. Note that other factors in the microenvironment of the virus, such as temperature and viscosity of the surrounding fluid, will also affect the diffusion coefficient as described by the Stokes-Einstein equation [82]. However, differences in temperature and viscosity in the in vitro environments of RSV and influenza virus are smaller than the difference in their size, so size is the most likely contributor to differences in the diffusion coefficient.

While differences in diffusion coefficient might play a role in increasing infecting time in vitro, influenza virus is known to be pleomorphic, with spherical forms dominating lab strains of the virus and filamentous forms more ubiquitous in vivo [83, 84]. The diffusion coefficient of rods is less than that of spheres [85], possibly equalizing the diffusion rates of RSV and influenza virus in vivo. Additionally, motion of influenza virus is known to be affected by neuraminidase activity [79, 86, 87] which is altered as influenza virus changes from spherical to filamentous morphology [88]. Influenza virus binds to sialic acids found on mucins, preventing it from moving. These bonds are broken by neuraminidase, allowing influenza virus to return to its normal motion. Thus the more neuraminidase activity, the faster influenza virus will move through its surroundings.

The infecting time also includes entry of the virus into a new target cell. For influenza virus, the time for initial fusion of the hemagglutinin protein to the viral membrane is approximately 20–30 s with full pore formation occurring 10–30 s later [89–91], but similar measurements have not yet been made for RSV. While we are lacking some measurements of the duration of processes that contribute to the infecting time, the difference in diffusion coefficients between RSV and influenza virus could partially explain the differences in infecting times measured in this study. More detailed measurements of the time needed for RSV to enter a host cell, together with studies of intercellular virion motion are needed to form a complete picture of the mechanisms leading to the observed differences in infecting time.

### Other differences between RSV and influenza virus

There are a number of key differences in the life cycles of RSV and influenza virus that could also contribute to the differences we have found. An important biological difference between influenza virus and RSV is RSV’s ability to form syncytia [92–94], although syncytia are much less observed in vivo than in vitro [93]. This feature is not explicitly included in our model, so its effect will be implicitly contained in the estimated values of the parameters. The effect of the syncytia is, in part, to alter the mode of transmission of virus, allowing them to move directly from cell to cell [95]. Differences in mode of transmission of the virus could also affect the observed infecting time.

Influenza virus and RSV also target different cells in the respiratory tract. RSV primarily targets ciliated cells in the bronchiolar and alveolar epithelium [47, 96–98], while human strains of influenza virus target primarily the non-ciliated cells [99]. These differences in cell tropism are caused by underlying differences in the distributions of receptors responsible for attachment for each of these viruses [100]. While cell type will not directly affect the time it takes for virions to move from one cell to another, it could alter the time it takes for a virion to attach to and fuse with the cell membrane, which would alter the infecting time. Additionally, the density of different cell types varies in the respiratory tract with studies indicating that the epithelium of the upper airway comprises 50–85% nonciliated cells [101, 102]. This means that the primary target cells for influenza virus are likely closer together than the primary target cells for RSV which would also lead to a lower infecting time for influenza virus. Interestingly, phases of the RSV and influenza virus life cycle that seem to be more dependent on cell type, such as the duration of the eclipse or infectious phases, are not consistently different between RSV and influenza virus. More complex models that include intracellular processes would aid to differentiate between RSV and influenza virus, but more detailed data is needed to accurately describe these processes [26, 103, 104].

Not only do influenza virus and RSV differ in the cells they target, but their cytopathic effects differ. A number of studies have noted that RSV has little cytopathic effect [97, 98, 105] in vitro, although in vivo columnar cell cytopathology and shedding of cellular debris seems to be a cause of bronchiolitis in young children [106]. Influenza virus, on the other hand, seems to be highly pathogenic in cells of the respiratory tract [107, 108]. Differences in cytopathology would likely manifest themselves in model parameters characterizing later stages of the infection cycle such as the duration of the infectious phase. We do see some statistically significant differences in duration of the infectious phase between RSV and influenza virus, although it is not consistent across all strains of influenza virus.

In humans, unlike in vitro systems where there are static and limited innate immune responses (IR) present, the complex cellular, adapted and innate IRs in vivo could also lead to differences in infection dynamics between RSV and influenza virus. Several differences in the immune responses to influenza virus and RSV have already been observed. In vitro, experimental infection of A549 cells with either virus showed similar type I IFN responses [9]. However, experimental infection of lymphocytes and macrophages with either virus has resulted in different IFN responses [109, 110], and different interleukin responses [111, 112]. Differences between the immune response to RSV and influenza in vivo have also been observed such as differences in which cytokines are secreted [10], in the interferon response [9, 113], in the activation and proliferation of lymphocytes [114], and in the movement of dendritic cells and monocytes to the respiratory tract [11]. These different immune responses, particularly differences in the early innate immune response, could contribute to the observed differences in growth rate and time of peak. The extent to which immunological differences alter dynamics between the two diseases is something that needs further investigation, using experiments in the same cell line in vitro and animal models for direct comparison of the two infections.

### Limitations of the data

The data used in this analysis was collected from a variety of previously published experiments and so lacks consistency. It is known that viral kinetics parameters can vary substantially between experiments, even when the same virus and cell line is used and the experiment is done in the same lab [21], so our use of data from experiments done in different labs is not ideal. For example, the RSV experiments collected here are performed in different cell types. While our comparison of WSN33 viral kinetics parameters in MDCK and MDBK cells suggests that there is little change in our parameter estimates when cell type changes, other experiments have seen very different dynamics using other strains of influenza virus in these two types of cells [72–74]. Further experiments with influenza virus in other cell lines are needed to properly assess the effect of cell type. We also need to be careful in extrapolating these results to RSV where virus-cell interactions are different and might be more sensitive to changes in cell type. Experiments directly comparing the viral kinetics of RSV in different cell lines would allow us to determine how much using data from different cell lines has contributed to the variability of our measurements. To perform a fair comparison of RSV and influenza virus, we would ideally like to have data from infections of both viruses in the same type of cells. Since many influenza virus in vitro experiments are performed in MDCK cells, experimental infection of these cells with RSV would allow for a good comparison of viral kinetics parameters. RSV is known to infect MDCK cells [115–118], so this would provide a consistent substrate for comparing influenza virus and RSV.

Our use of data from specific RSV and influenza virus strains might not be reflective for all strains. It is known that the RSV A2 strain differs in viral load and pathology from other RSV strains [119, 120]. It is also demonstrated that variations in the amino acid sequence of envelope proteins may contribute to differences in infection time. It has been shown for instance that drug-induced mutations in RSV F seem to result in differences in viral infection rate [121, 122]. Moreover, two studies investigating the fitness of an influenza virus drug-resistant strain demonstrated that although the strain seemed to display equal fitness as compared to the wild-type strain, the mutation caused subtle differences in the viral kinetics, lengthening one phase but shortening another, such that the net effect is that there is little discernible difference in the viral titers of the two influenza virus strains [20, 21].

A further limitation of this study is the limited amount of information in each individual data set. While several of the viral kinetics parameters have been previously estimated for in vitro influenza virus infections, some of our values do not agree well with these estimates. The infecting time was estimated to be 0.08–1.5 h in several different in vitro experiments using different influenza virus strains [20, 37, 123, 124]. Our median estimates all lie within this range. Previous estimates of the duration of the eclipse phase obtained in vitro range from 4–11 h [20, 21, 35, 123, 125, 126]. Our median estimates are quite a bit shorter than previous results. The duration of the infectious phase for influenza virus has also been measured before, with estimates ranging from 10.5–49 h [20, 21, 35, 37], which are again longer than our median estimates for *τ*_*I*_. This discrepancy is likely due to identifiability issues particularly for the viral kinetics model. For the empirical model, which has four free parameters, two of the data sets only had four data points, limiting the reliability of the parameter estimates. The situation is even worse for the viral kinetics model, which has six free parameters. In this case, 22 of the 36 data sets used here had 6 or fewer data points, meaning that some of the parameter estimates are unreliable. This could be the cause of the broad distributions of parameter estimates we find for *τ*_*E*_ and *τ*_*I*_, leading to difficult in measuring significant differences in these quantities. It is well-known that viral time course alone will not allow for unique identification of all the parameters in the viral kinetics model [39, 127, 128]. Additional data from single-cycle experiments [20, 21], or from measurements of RNA [128] will help improve confidence in our parameter estimates. Even simple improvements such as measuring more often for a longer time will increase reliability of our estimates. While we limited our data sets to those that contained both growth and decay of virus, several of these experiments had only two points in one of those phases. With the inherent error in viral titer measurements [129, 130], the limited number of data points in each time course leads to error in the viral kinetics parameter estimates.

## Conclusion

In summary, our analysis has found differences between influenza virus and RSV dynamics in vitro that are consistent with observed differences in influenza virus and RSV dynamics in vivo [12]. Our finding of differences in infecting time suggests a possible mechanism at the virus-cell level for the differences observed in vivo and in vitro. The mechanism of different infecting times is supported by known differences in the diffusion rates of the two viruses, although this is not the only factor that determines infecting time. More consistent experiments, as suggested in the discussion, should further help develop our understanding of the differences in RSV and influenza virus dynamics.
